# Improves symptoms and urinary biomarkers in refractory interstitial cystitis/bladder pain syndrome patients randomized to extracorporeal shock wave therapy versus placebo

**DOI:** 10.1038/s41598-021-87040-1

**Published:** 2021-04-06

**Authors:** Yuan-Chi Shen, Pradeep Tyagi, Wei-Chia Lee, Michael Chancellor, Yao-Chi Chuang

**Affiliations:** 1 Department of Urology, Kaohsiung Chang Gung Memorial Hospital, Chang Gung University College of Medicine, 123 Ta Pei Road, Niao Song District, Kaohsiung, Taiwan; 2 The Center of Excellence in Shockwave Medicine and Tissue Regeneration, Kaohsiung Chang Gung Memorial Hospital, Chang Gung University College of Medicine, 123 Ta Pei Road, Niao Song District, Kaohsiung, Taiwan; 3 Department of Urology, School of Medicine, University of Pittsburgh, Pittsburgh, PA USA; 4 Department of Urology, Beaumont Health System, Oakland University William Beaumont School of Medicine, Royal Oak, MI USA

**Keywords:** Biomarkers, Diseases, Urology

## Abstract

Extracorporeal shock wave therapy (ESWT) has been shown to improve symptoms in patients with interstitial cystitis/bladder pain syndrome (IC/BPS); however, there is a lack of objective evidence. We measured change of urinary biomarker levels in 25 patients with IC/BPS received ESWT or placebo once a week for 4 weeks. Urines were collected from participants at baseline, 4 and 12 weeks post treatment. A representative 41 inflammatory growth factors, cytokines, and chemokines in urine were measured using a MILLIPLEX immunoassay kit. Symptom bother was assessed by O’Leary-Sant symptom scores (OSS), and visual analog scale (VAS) for pain. The ESWT group exhibited a significant reduction in the OSS and VAS compared to the placebo group 4 weeks post-treatment (*P* < 0.05), and the effects were persistent at 12 weeks. The difference in urinary markers change in ESWT versus placebo was *P* = 0.054 for IL4, *P* = 0.013 for VEGF, and *P* = 0.039 for IL9 at 4 weeks. The change of urine biomarker was not significant in other biomarkers or all the measured proteins at 12 weeks. The current data suggest that IL4, IL9, and VEGF mediation may be involved in its pathophysiologic mechanisms and response to LESW treatment.

## Introduction

Interstitial cystitis/bladder pain syndrome (IC/BPS) is a chronic disease characterized by symptoms of unpleasant sensation, pain, pressure, and discomfort perceived to be related to the urinary bladder, associated with lower urinary tract symptoms, in the absence of infection or other identifiable causes^1^. Treatment of refractory IC/BPS patients includes hydrodistension, oral medications, and intravesical therapy has met with only limited efficacy and there is an unmet need for developing new therapy for IC/BPS^2^.

The etiology and pathogenesis of IC/BPS are multifactorial and several studies have attributed to the exhibited symptoms to an increase in inflammatory cells infiltration, hypoxia-inducible factor-1α, VEGF, and apoptosis^3,4^ noted on histopathological and molecular studies. Thus, the treatment of IC/BPS aiming at regulation of inflammatory reaction and regeneration or repair of urothelium defect could be an attractive option for the management of refractory IC/BPS.

Low energy shock wave (LESW), known to exert anti-inflammatory, anti- apoptotic effects, and improve tissue repair, has been applied for the treatment of urological disease, including erectile dysfunction, and chronic prostatitis chronic pelvic pain syndrome^5,6^. Furthermore, recent publications have been extended into the field of bladder dysfunction and demonstrated therapeutic effects of LESW on overactive bladder and IC/BPS^7,8^. The approaches used for mechanistic studies on efficacy of LESW in animal model^6,9^ need to be modified for human confirmation . Towards that end we assessed the change in urine levels of multiple growth factors, cytokines, chemokines, and clinical symptoms of IC/BPS patients at baseline and after LESW or placebo treatment.

## Materials and methods

### Study design

The Urinary Marker study was a planned exploratory supplementary study to the phase II trial, Low Energy Shock Wave (LESW) for the treatment of IC/BPS—a, randomized, double-blind, placebo-controlled, prospective study (ClinicalTrials.gov number, NCT03619486). Details of the trial design and methods have been previously published^8^.

The current study only included patient population from one center (KCGMH) for urinary marker analysis. The IC/BPS confirmed patients were randomly assigned to receive (1) LESW or (2) placebo group in a 1:1 ratio after the review and approval by the Institutional Review Board of the hospital (Chang Gung Memorial Hospital IRB 201800525A3), and was in compliance with the ethical principles of Good Clinical Practice guidelines and the principles of the Declaration of Helsinki. Informed consent was obtained from patients before any study procedures were performed.

### Study population

Patients with IC/BPS, who aged 20 years or above and had failed at least 6 months of conventional treatments, including non-steroidal anti-inflammatory drugs (NSAIDs), hydrodistension, intravesical hyaluronic acid instillation, or intravesical botulinum toxin A injection, were enrolled. The diagnosis of IC/BPS was established based on characteristic symptoms of unpleasant sensation (pain, pressure, discomfort) perceived to be related to the urinary bladder of more than 6 months duration, in the absence of infection or other identifiable causes and cystoscopic findings of glomerulations, petechia, or mucosal fissures upon hydrodistention under anesthesia to 80 cm H_2_O pressure for 3 min.

They had no evidence of active urinary tract infection, neurogenic bladder dysfunction, or coagulopathy. The inclusion and exclusion criteria are listed in the Appendix of our previous publication^8^. Permuted block randomization method was applied to generate randomization codes. Each randomization number was assigned to individual patient according to the time-sequence for screened patient become eligible.

### Treatment

The procedures were done by one experienced urologist as an office procedure without any anesthesia. Studied patients were placed in a supine position with bladder distended with up to 50–100 cc of urine volume as detected by transabdominal ultrasonography. The shock wave applicator (LITEMED LM ESWT mini system, Taiwan) or placebo applicator were gently placed directly on the ultrasound transmission gel over the skin surface of suprapubic region above the urinary bladder at the range of transverse crease 2–4 cm above the pubic bone and 4 cm width, once a week for 4 weeks, with 2000 shocks, frequency of 3 pulses per second, and maximum energy flow density 0.25 mJ/mm^2^^8^. The device used for the study was a standard electromagnetic shock wave unit with a focus zone penetration depth in the range of 20–150 mm, which meant that this wide focused shock wave could be placed in the bladder from the suprapubic area easily^8^. The placebo treatment was performed with the therapy head of the same outward appearance, which was also fitted with a placebo stand-off without energy transmission.

Follow-up visits were scheduled at 1 week, 4 weeks, and 3 months post-treatment. The blinding included the specification that neither the patient nor the investigator/follow-up observer was aware of placebo or ESWT assignment. In the event of inadequate pain relief or worsening IC symptoms during the study period, patients were permitted to take acetaminophen.

### Urine processing

Urine samples were collected at baseline, post treatment 4 weeks and 12 weeks at KCGMH. Specimens were kept on ice or at 4° C for short times until stored at − 80 °C (within 2–4 h). The urine was centrifuged (12,000 rpm × 15 min) at 4 °C, and the supernatants were directly analyzed.

### Multiplex analysis

The urine sample were stored at − 80 °C until analysis by MILLIPLEX MAP Human Cytokine/Chemokine Panel (Merck Millipore, Billerica, MA), a magnetic bead-based immunology multiplex assay, which can simultaneously quantify the following 41 human cytokines: sCD40L, EGF, FGF-2, Flt-3 ligand, Fractalkine, G-CSF, GM-CSF, GRO, IFN-α2, IFN-γ, IL-1α, IL-1β, IL-1RA, IL-2, IL-3, IL-4, IL-5, IL-6, IL-7, IL-8, IL-9, IL-10, IL-12 (p40), IL-12 (p70), IL-13, IL-15, IL-17A, IP-10, MCP-1, MCP-3, MDC (CCL22), MIP-1α, MIP-1β, PDGF-AB/BB, RANTES, TGF-α, TNF-α, TNF-β, VEGF, Eotaxin/CCL11, PDGF-AA. The samples were processed in duplicate according to the manufacturer’s instructions^10^. Cytokine concentrations were normalized to urine creatinine content.

### Outcome measures

The average changes in O’Leary-Sant symptom scores (OSS), a 3-day voiding diary, Visual Analog Pain Scale (VAS, 0–10), global response assessment (GRA) with categorizations (− 3, − 2, − 1, 0, 1, 2, 3), uroflowmetry, and residual urine detected by ultrasonography at 1, 4, and 12 weeks were compared to baseline and between groups after treatment.

### Statistical methods

The average change in values from baseline at 1, 4, and 12 weeks post-treatment in studied parameters, scores or outcome measures, and net changes of each efficacy item between treatment group and the controlled groups were analyzed using generalized estimating equation. The patient-responded global assessment was analyzed using Fisher’s exact test between the treatment and the controlled groups. All statistical assessments were considered significant at *P* < 0.05. Statistical analyses were performed using SPSS version 22.0 statistical software (SPSS Inc., Chicago, IL).

## Results

### Patient disposition

**Figure 1:**
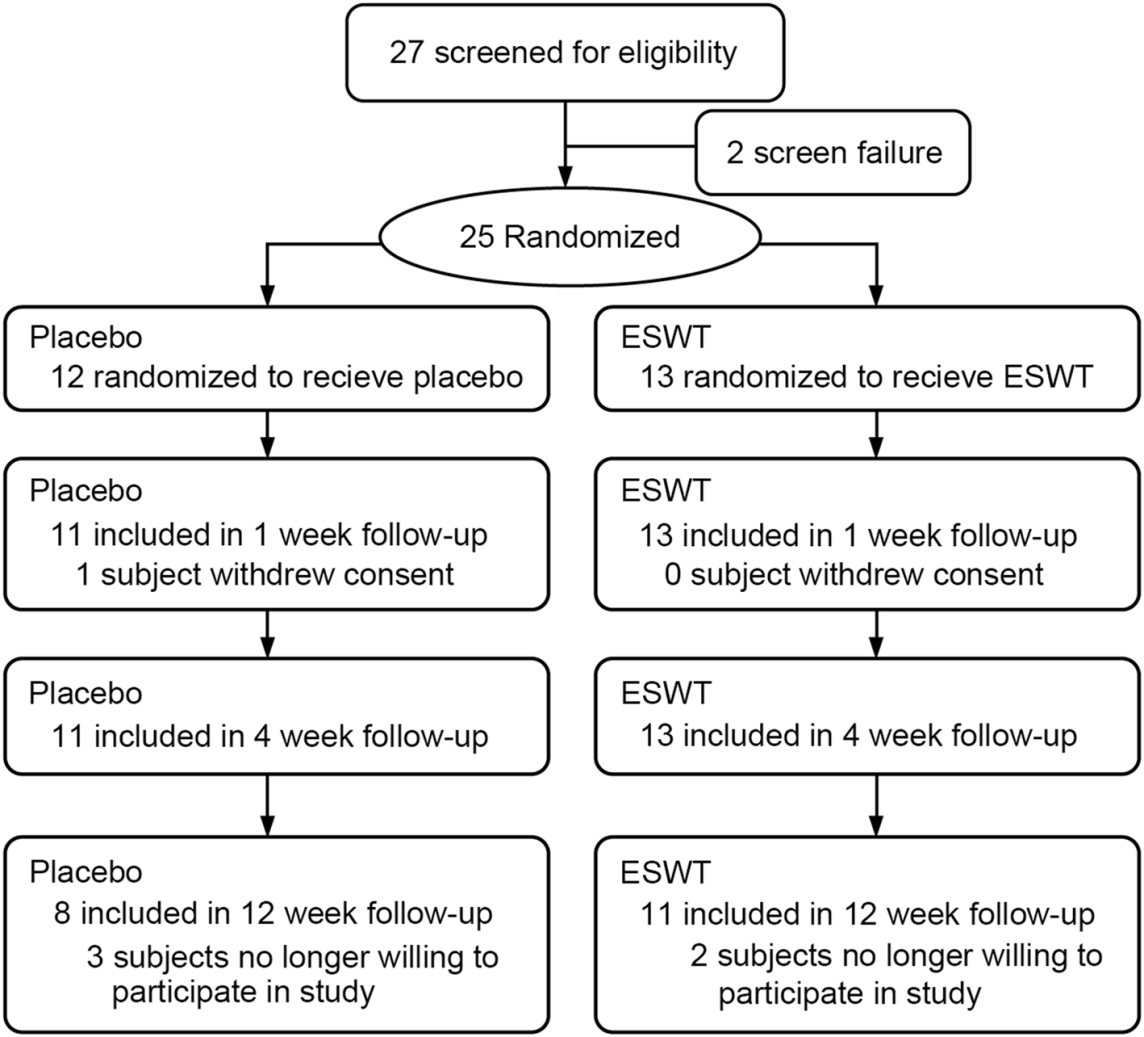
Patient allocation and flow chart of the study.

A total of 27 patients were screened and 25 eligible patients were randomly allocated into two subgroups. One patient withdrew consent and thereby did not receive any treatment (Fig. 1). The final intent-to-treat population consisted of 24 subjects, including 13 in ESWT, and 11 in placebo groups of whom all subjects completed the primary endpoint evaluation (i.e. 4 weeks post-treatment). The baseline characteristics were comparable across the treatment groups except pain and voided volume, which was significant less in the placebo group (Tables 1 and 2). None of the participants had Hunner lesions, but all of them showed some evidence of bladder inflammation from pathological findings (supplementary file). One patient was associated with fibromyalgia and Sicca syndrome in the ESWT group, and one patient was associated with Sicca syndrome and irritable bowel syndrome in the placebo group.

**Table 1:** Baseline patient characteristics.

Variable	Placebo (n = 12)	ESWT (n = 13)	*P* value^a^
	Mean (95%CI)	Mean (95%CI)	
Age	57.8 (49.5, 66.0)	55.7 (48.3, 63.1)	0.687
No. male/female	5/6	3/10	0.340
Anesthetic bladder capacity (ml)^b^	618.6 (521.0, 716.2)	677.0 (595.6, 758.4)	0.322
Duration of disease (yr)	5.8 (4.2, 7.3)	6.2 (3.6, 8.8)	0.758

Values are presented as mean (95%CI).

*P < 0.05, **P < 0.01, ***P < 0.005, ****P < 0.0001.

^a^Comparison between ESWT and placebo (Two sample t-test).

^b^During hydrodistention.

**Table 2:** Variables at baseline, 1, 4, and 12 weeks in patients in ESWT and placebo.

Variable	Placebo (N = 12)		ESWT (N = 13)		*P* value^b^
	Mean (95% CI)	p value ^a^	Mean (95% CI)	*P* value^a^	
**O' Leary-Sant score**					
Baseline	25.6 (22.5, 28.6)		26.0 (22.6, 29.4)		0.834
Wk 1	23.6 (19.8, 27.3)	0.061	20.4 (16.9, 23.9)	< 0.0001****	
Wk 4	24.8 (21.3, 28.3)	0.512	20.6 (16.6, 24.6)	< 0.0001****	
Wk 12	25.1 (20.7, 29.6)	0.061	18.2 (13.2, 23.1)	< 0.0001****	
**ICSI**					
Baseline	12.3 (10.1, 14.5)		12.7 (10.6, 14.8)		0.789
Wk 1	10.8 (8.1, 13.6)	0.056	9.3 (7.1, 11.5)	0.001***	
Wk 4	11.7 (8.9, 14.6)	0.511	9.2 (6.9, 11.5)	< 0.0001****	
Wk 12	11.8 (8.4, 15.2)	0.958	7.9 (5.0, 10.9)	< 0.0001****	
**ICPI**					
Baseline	13.3 (11.5, 15.0)		13.3 (11.8, 14.8)		0.954
Wk 1	12.7 (11.1, 14.4)	0.518	11.1 (9.4, 12.7)	0.001***	
Wk 4	13.1 (11.8, 14.4)	0.795	11.2 (9.4, 13.1)	0.001***	
Wk 12	13.3 (11.5, 15.1)	0.567	10.2 (8.0, 12.5)	0.001***	
**VAS**					
Baseline	5.3 (4.2, 6.3)		6.9 (5.8, 8.0)		0.017*
Wk 1	5.1 (4.0, 6.2)	0.513	4.9 (3.4, 6.3)	< 0.0001****	
Wk 4	4.8 (4.0, 6.1)	0.192	4.9 (3.3, 6.4)	< 0.0001****	
Wk 12	5.2 (3.1, 7.3)	0.862	4.7 (3.0, 6.4)	< 0.0001****	
**FBC (ml)**					
Baseline	282.1 (197.3, 366.8)		315.4 (258.0, 372.8)		0.456
Wk 1)	238.9 (163.5, 314.3)	0.037*	287.7 (231.9, 343.5)	0.161	
Wk 4	294.6 (194.9, 394.2)	0.381	296.9 (225.8, 368.0)	0.359	
Wk 12	250.6 (142.0, 359.2)	0.222	293.2 (218.5, 367.8)	0.177	
**Frequency**					
Baseline	11.6 (7.7, 15.5)		11.1 (8.8, 13.4)		0.793
Wk 1	12.4 (9.0, 15.8)	0.376	10.2 (7.7, 12.7)	0.088	
Wk 4	12.0 (8.5, 15.8)	0.908	10.4 (7.2, 13.6)	0.331	
Wk 12	12.6 (7.2, 18.1)	0.022*	9.8 (6.7, 13.0)	0.005**	
**Nocturia**					
Baseline	2.6 (1.4, 3.8)		1.7 (0.7, 2.8)		0.196
Wk 1	2.9 (1.5, 4.3)	0.243	1.7 (0.7, 2.6)	0.792	
Wk 4	2.3 (1.0, 3.6)	0.380	1.6 (0.6, 2.7)	0.780	
Wk 12	3.9 (1.4, 6.4)	0.125	1.8 (0.7, 0.9)	0.889	
**Qmax (ml/s)**					
Baseline	14.4 (8.5, 20.2)		16.3 (12.0, 20.7)		0.533
Wk 1	17.5 (9.9, 25.2)	0.373	16.8 (13.4, 20.3)	0.663	
Wk 4 (N = 11, 13)	17.6 (10.5, 24.6)	0.329	17.1 (11.9, 22.4)	0.705	
Wk 12 (N = 8, 11)	21.1 (11.5, 30.6)	0.290	16.0 (12.9, 19.1)	0.873	
**Voided volume (ml)**					
Baseline	172.0 (112.1, 231.8)		256.9 (187.5, 326.4)		0.034*
Wk 1	185.0 (129.2, 240.8)	0.835	277.0 (199.4, 354.6)	0.316	
Wk 4	238.6 (150.0, 327.1)	0.075	248.8 (163.4, 334.3)	0.792	
Wk 12	243.1 (177.2, 308.9)	0.106	320.6 (210.8, 430.4)	0.038*	
**Residual urine (ml)**					
Baseline	25.0 (7.7, 42.4)		33.8 (17.0, 50.5)		0.408
Wk 1	32.1 (10.6, 53.6)	0.680	25.5 (15.7, 35.2)	0.303	
Wk 4	31.7 (9.1, 54.4)	0.415	28.8 (5.6, 51.9)	0.409	
Wk 12	31.3 (6.1, 56.5)	0.590	38.5 (13.7, 63.3)	0.748	
**GRA (< 2/ ≥ 2)**					
Wk 1	9/2 (18.2%)		6/7 (53.8%)		0.105^c^
Wk 4	8/3 (27.3%)		6/7 (53.8%)		0.240^c^
Wk 12	8/1 (11.1%)		6/7 (53.8%)		0.074^c^

Values are presented as mean (95% CI).

*FBC* functional bladder capacity, the maximal voided volume that appears in the three-day voiding diary, *Voided volume* obtained from uroflowmetry to test the amount of urine voided during urination.

*P < 0.05, **P < 0.01, ***P < 0.005, ****P < 0.0001.

^a^Generalized estimating equation compared with baseline.

^b^Comparison between corresponding time point of ESWT and placebo (generalized estimating equation).

^c^Comparison between corresponding time point of ESWT and placebo (Fisher's exact test).

### Efficacy and safety

**Figure 2:**
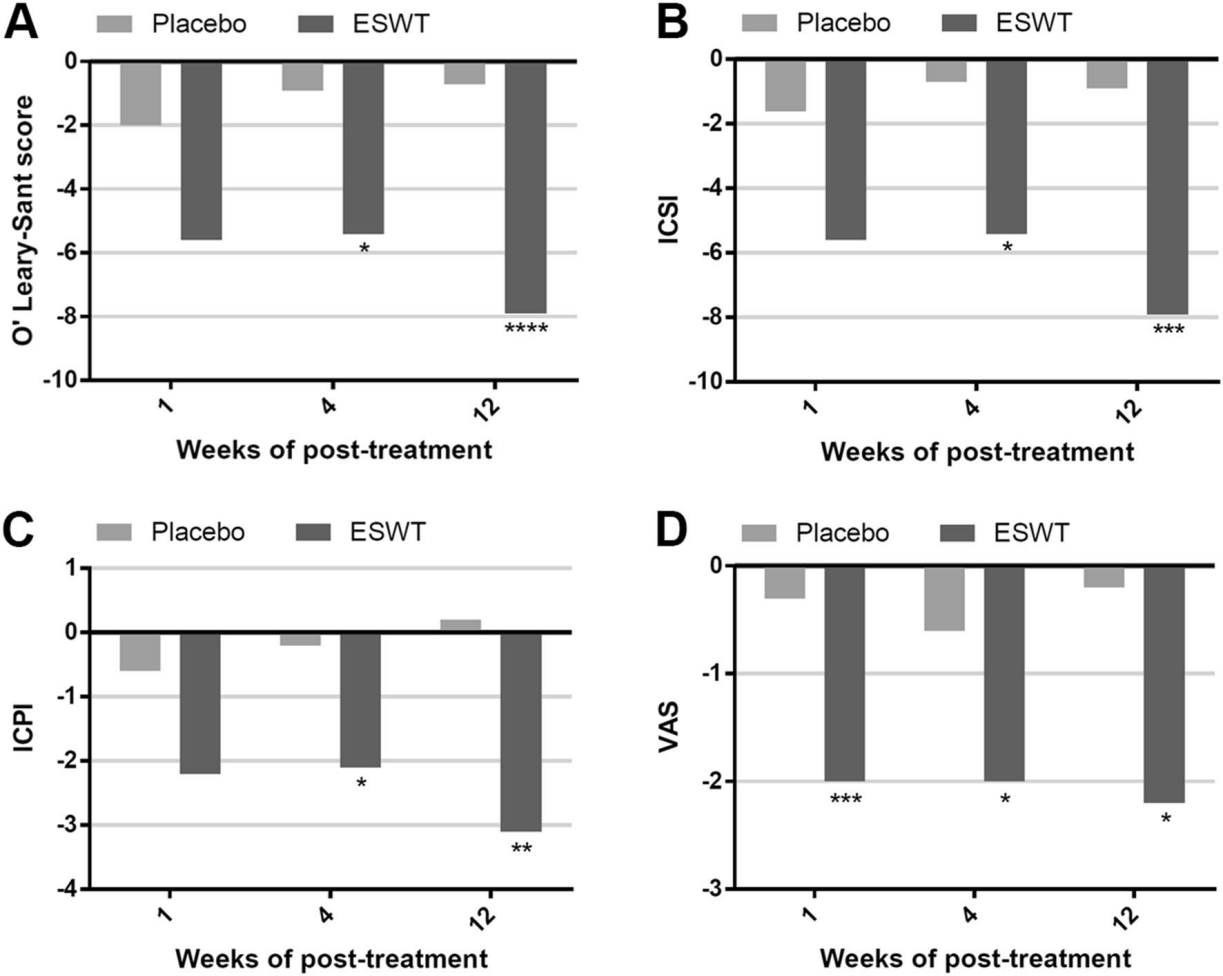
Outcome measures of ESWT group versus placebo group at baseline, week 1, week 4 and week 12 in OSS (**A**), ICSI (**B**), ICPI (**C**) and VAS (**D**). *ESWT* extracorporeal shock wave therapy, *ICPI* interstitial cystitis problem indices, *ICSI* interstitial cystitis symptom indices, *OSS* O'Leary‐Sant symptom scores, *VAS* visual analog scale. *P < 0.05, **P < 0.01, ***P < 0.005, ****P < 0.0001.

Statistically significant improvements occurred in the pain scale and OSS, including ICSI and ICPI, from baseline to 1 week, 4 weeks and 12 weeks after treatment in ESWT group (Table 2; Fig. 2) compared to an insignificant change in the placebo group for OSS, ICSI, ICPI, and VAS. The difference in improvement between ESWT versus placebo for OSS was − 5.4 (− 8.3, − 2.5) versus − 0.9 (− 4.0, 2.2) (95% CI; *P* = 0.014) at 4 weeks and the efficacy was maintained till 12 weeks (Fig. 2). The difference in VAS improvement was − 2.0 (− 3.1, − 0.9) versus − 0.6 (− 1.4, 0.3) (95% CI; *P* = 0.018) at 4 weeks, and the efficacy lasted till 12 weeks. No patient had urinary incontinence, retention or infection with ESWT or placebo treatment.

### Urine biomarkers

Of the 41 candidate markers, 19 markers were measurable in urine and analyzed. Biomarkers levels at baseline, 4 and 12 weeks following treatment are shown in Table 3. No baseline differences were found between ESWT and placebo groups. IL4, IFNα2, and VEGF levels were significantly increased at 4 weeks in placebo group, and IL9, and Flt3 levels were significantly increased at 4 weeks post ESWT treatment. The difference between ESWT versus placebo for the change in urinary markers was − 0.0 (− 0.3, 0.2) versus 0.4 (− 0.0, 0.9) (95% CI; IL4, *P* = 0.054) and − 0.5 (− 1.3, 0.7) versus 1.2 (0.3, 2.1) (95% CI; VEGF, *P* = 0.013), and 0.2 (− 0.0, 0.4) versus − 0.0 (− 0.1, 0.0) (95% CI; IL9, *P* = 0.039) at 4 weeks (Table 3).

**Table 3:** Cytokine at baseline, 1, 4, and 12 weeks in patients in ESWT and placebo.

Cytokine (pg/mg of creatinine)	Placebo (N = 11)				ESWT (N = 13)				*P* value^c^	*P* value^d^
	Cytokine		Cytokine change		Cytokine		Cytokine change			
	Mean (95% CI)	*P* value^a^	Mean (95% CI)	*P* value^b^	Mean (95% CI)	*P* value^a^	Mean (95% CI)	*P* value^b^		
**IL-1RA**										
Baseline	7.9 (0.1, 15.7)				14.8 (− 4.2, 33.9)				0.493	
Wk 1	5.3 (1.1, 9.5)	0.685	− 4.3 (− 12.0, 3.5)	0.424	35.4 (− 26.2, 96.9)	0.305	20.6 (− 22.1, 63.2)	1.000		0.298
Wk 4	8.6 (2.7, 14.5)	0.921	0.7 (− 5.3, 6.6)	0.898	19.0 (− 3.3, 41.3)	0.936	3.4 (− 2.3, 9.1)	1.000		0.458
Wk 12	20.4 (− 14.7, 55.5)	0.015*	9.8 (− 12.8, 32.4)	0.079	40.7 (− 35.7, 117.0)	0.141	24.8 (− 28.6, 78.2)	1.000		0.632
**IL-4**										
Baseline	0.5 (0.0, 1.0)				0.5 (0.1, 0.9)				0.972	
Wk 1	0.5 (0.0, 1.1)	0.629	− 0.1 (− 0.7, 0.6)	0.772	0.3 (0.1, 0.5)	0.301	− 0.2 (− 0.6, 0.1)	0.138		0.577
Wk 4	0.9 (0.1, 1.7)	0.028*	0.4 (− 0.0, 0.9)	0.195	0.5 (0.1, 1.0)	0.776	− 0.0 (− 0.3, 0.2)	0.895		0.054
Wk 12	0.8 (− 0.2, 1.7)	0.760	0.0 (− 1.1, 1.1)	0.697	0.9 (0.2, 1.6)	< 0.0001****	0.3 (0.1, 0.6)	0.079		0.419
**IL-6**										
Baseline	0.2 (0.1, 0.2)				0.2 (0.1, 0.2)				0.685	
Wk 1	0.2 (0.1, 0.3)	0.834	− 0.0 (− 0.1, 0.1)	0.967	0.2 (0.1, 0.4)	0.241	0.0 (− 0.1, 0.1)	0.461		0.609
Wk 4	0.3 (− 0.1, 0.7)	0.214	0.2 (− 0.2, 0.6)	0.223	0.2 (0.1, 0.3)	0.746	0.0 (− 0.0, 0.1)	0.643		0.418
Wk 12	0.1 (0.0, 0.3)	0.462	0.0 (− 0.1, 0.1)	0.260	0.2 (0.2, 0.3)	0.168	0.1 (0.0, 0.1)	0.043*		0.087
**IL-8**										
Baseline	1.3 (− 0.4, 2.9)				1.1 (− 0.1, 2.2)				0.812	
Wk 1	0.3 (0.1, 0.4)	0.775	− 1.3 (− 3.3, 0.7)	0.662	2.8 (− 1.0, 6.6)	0.035*	1.8 (− 1.1, 4.6)	0.093		0.087
Wk 4	4.9 (− 4.5, 14.2)	0.302	3.6 (− 6.1, 13.3)	0.292	1.3 (− 0.2, 2.8)	0.970	0.2 (− 0.1, 0.5)	0.925		0.408
Wk 12	1.3 (− 1.1, 3.7)	0.988	0.6 (− 1.6, 2.8)	0.976	1.5 (− 1.0, 4.0)	0.928	0.3 (− 1.0, 1.7)	0.978		0.794
**IL-9**										
Baseline	0.2 (0.1, 0.3)				0.4 (0.2, 0.5)				0.179	
Wk 1	0.2 (0.1, 0.3)	0.255	− 0.0 (− 0.2, 0.1)	0.478	0.5 (0.2, 0.7)	0.005**	0.1 (− 0.1, 0.3)	0.304		0.190
Wk 4	0.2 (0.2, 0.2)	0.322	− 0.0 (− 0.1, 0.0)	0.546	0.5 (0.3, 0.8)	0.002***	0.2 (− 0.0, 0.4)	0.054		0.039*
Wk 12	0.2 (0.0, 0.3)	0.070	− 0.0 (− 0.2, 0.2)	0.409	0.4 (0.3, 0.5)	0.478	0.0 (− 0.1, 0.2)	0.885		0.635
**IL-10**										
Baseline	0.3 (0.1, 0.4)				0.3 (0.2, 0.4)				0.864	
Wk 1	0.3 (0.1, 0.4)	0.930	− 0.0 (− 0.3, 0.2)	1.000	0.3 (0.1, 0.5)	0.572	0.1 (− 0.2, 0.3)	0.523		0.591
Wk 4	0.3 (0.2, 0.4)	0.221	0.1 (− 0.0, 0.2)	1.000	0.3 (0.2, 0.5)	0.557	0.1 (− 0.0, 0.2)	0.619		0.693
Wk 12	0.3 (0.0, 0.6)	0.896	0.0 (− 0.3, 0.4)	1.000	0.5 (0.3, 0.7)	0.003***	0.3 (0.1, 0.4)	0.020*		0.087
**IL-15**										
Baseline	0.3 (0.2, 0.5)				0.3 (0.2, 0.4)				0.928	
Wk 1	0.3 (0.2, 0.5)	0.902	− 0.0 (− 0.2, 0.2)	0.877	0.4 (0.2, 0.5)	0.722	0.0 (− 0.1, 0.2)	0.674		0.702
Wk 4	0.4 (0.2, 0.5)	0.543	0.0 (− 0.1, 0.2)	0.704	0.4 (0.2, 0.6)	0.345	0.1 (− 0.0, 0.3)	0.372		0.334
Wk 12	0.3 (0.1, 0.6)	0.402	− 0.0 (− 0.3, 0.3)	0.693	0.5 (0.3, 0.8)	0.003***	0.3 (0.1, 0.5)	0.024*		0.065
**FGF2**										
Baseline	3.7 (0.7, 6.8)				3.7 (1.6, 5.7)				0.956	
Wk 1	2.5 (1.4, 3.7)	0.126	− 1.3 (− 4.6, 1.9)	0.209	4.6 (1.6, 7.7)	0.556	1.0 (− 2.6, 4.6)	0.539		0.315
Wk 4	4.3 (2.6, 6.0)	0.275	0.5 (− 1.4, 2.5)	0.891	4.0 (1.8, 6.2)	0.909	1.3 (− 0.7, 3.3)	0.840		0.568
Wk 12	3.3 (0.7, 5.9)	0.476	− 0.4 (− 4.4, 3.7)	0.561	5.7 (2.2, 9.1)	0.091	3.1 (0.3, 5.9)	0.207		0.092
**G-CSF**										
Baseline	0.8 (0.4, 1.2)				1.0 (0.2, 1.7)				0.781	
Wk 1	0.7 (0.2, 1.2)	0.467	− 0.3 (− 0.8, 0.2)	0.394	0.8 (0.4, 1.2)	0.710	− 0.2 (− 1.0, 0.7)	0.717		0.785
Wk 4	1.7 (0.3, 3.1)	0.061	0.8 (− 0.4, 2.1)	0.109	1.3 (0.2, 2.4)	0.398	0.4 (− 0.9, 1.6)	0.554		0.464
Wk 12	0.9 (0.1, 1.8)	0.193	− 0.1 (− 1.2, 0.9)	0.108	1.5 (0.7, 2.3)	0.094	0.5 (− 0.2, 1.2)	0.510		0.252
**Flt3**										
Baseline	0.8 (0.4, 1.3)				0.8 (0.3, 1.3)				0.963	
Wk 1	0.9 (0.4, 1.5)	0.635	− 0.1 (− 0.5, 0.3)	0.460	0.9 (0.4, 1.3)	0.224	0.0 (− 0.2, 0.3)	0.761		0.539
Wk 4	1.0 (0.3, 1.6)	0.355	0.1 (− 0.3, 0.5)	0.789	1.0 (0.4, 1.6)	< 0.0001****	0.2 (− 0.2, 0.5)	0.271		0.814
Wk 12	1.3 (0.3, 2.3)	0.313	0.1 (− 0.6, 0.8)	0.898	1.0 (0.4, 1.6)	0.005**	0.1 (− 0.3, 0.5)	0.505		0.986
**Fractalkine**										
Baseline	8.1 (2.8, 13.4)				7.0 (3.3, 10.7)				0.708	
Wk 1	7.9 (4.3, 11.6)	0.705	− 1.0 (− 6.8, 4.8)	0.668	6.9 (2.5, 11.2)	0.946	− 0.2 (− 6.0, 5.6)	0.949		0.827
Wk 4	10.0 (5.2, 14.7)	0.316	1.9 (− 3.7, 7.5)	0.468	6.1 (3.3, 8.9)	0.647	0.2 (− 3.5, 3.9)	0.634		0.568
Wk 12	6.6 (− 0.1, 13.3)	0.115	− 2.3 (− 6.5, 2.0)	0.584	8.4 (4.1, 12.7)	0.391	2.6 (− 2.0, 7.2)	0.582		0.097
**IFNα2**										
Baseline	0.9 (0.3, 1.5)				1.2 (0.5, 2.0)				0.512	
Wk 1	0.7 (0.4, 1.1)	0.678	− 0.2 (− 1.0, 0.5)	0.237	1.1 (0.4, 1.8)	0.835	− 0.1 (− 1.2, 1.0)	1.000		0.866
Wk 4	1.7 (0.7, 2.7)	0.046*	0.8 (− 0.3, 1.9)	0.245	1.0 (0.5, 1.4)	0.608	− 0.0 (− 0.8, 0.7)	1.000		0.171
Wk 12	0.9 (0.1, 1.8)	0.561	− 0.0 (− 1.0, 0.9)	0.312	1.4 (0.6, 2.1)	0.475	0.4 (− 0.6, 1.3)	1.000		0.533
**GRO**										
Baseline	1.2 (0.3, 2.0)				0.9 (0.4, 1.5)				0.595	
Wk 1	0.8 (0.3, 1.4)	0.233	− 0.6 (− 1.3, 0.1)	0.230	0.8 (0.3, 1.4)	0.842	− 0.1 (− 0.7, 0.5)	0.830		0.236
Wk 4	1.7 (0.2, 3.2)	0.183	0.5 (− 0.8, 1.9)	0.326	1.4 (0.1, 2.7)	0.214	0.5 (− 0.7, 1.6)	0.319		0.921
Wk 12	1.4 (0.1, 2.6)	0.051	− 0.3 (− 1.3, 0.7)	0.110	1.5 (0.6, 2.4)	0.156	0.5 (− 0.1, 1.0)	0.406		0.074
**MCP-1**										
Baseline	23.6 (11.3, 36.0)				26.1 (15.2, 36.9)				0.744	
Wk 1	26.1 (11.7, 40.4)	0.693	1.1 (− 14.8, 16.9)	0.383	28.4 (8.4, 48.3)	0.726	2.3 (− 13.5, 18.1)	0.770		0.906
Wk 4	18.1 (11.6, 24.6)	0.216	− 5.5 (− 14.7, 3.7)	0.835	30.2 (9.6, 50.7)	0.569	2.3 (− 12.4, 17.1)	0.655		0.330
Wk 12	16.8 (3.4, 30.3)	0.718	− 2.3 (− 20.9, 16.3)	0.857	25.1 (14.1, 36.1)	0.978	− 3.5 (− 17.1, 10.2)	0.711		0.906
**MCP-3**										
Baseline	1.3 (0.4, 2.2)				1.3 (0.7, 2.0)				0.873	
Wk 1	1.0 (0.6, 1.4)	0.432	− 0.3 (− 1.4, 0.7)	0.464	1.4 (0.6, 2.3)	0.864	0.1 (− 1.0, 1.2)	0.853		0.541
Wk 4	1.6 (1.0, 2.1)	0.125	0.3 (− 0.4, 1.0)	0.466	1.4 (0.7, 2.2)	0.823	0.4 (− 0.3, 1.0)	0.783		0.892
Wk 12	1.3 (0.3, 2.3)	0.662	− 0.1 (− 1.5, 1.4)	0.866	2.1 (0.9, 3.2)	0.033*	1.0 (0.1, 1.9)	0.130		0.122
**VEGF**										
Baseline	1.1 (0.5, 1.7)				1.5 (0.6, 2.3)				0.429	
Wk 1	1.5 (0.7, 2.4)	0.519	0.3 (− 0.4, 1.0)	0.539	1.1 (0.7, 1.5)	0.413	− 0.4 (− 1.3, 0.5)	0.415		0.241
Wk 4	2.3 (1.1, 3.4)	0.004***	1.2 (0.3, 2.1)	0.015*	1.2 (0.4, 2.0)	0.276	− 0.5 (− 1.3, 0.7)	0.394		0.013*
Wk 12	1.6 (0.4, 2.9)	0.960	0.3 (− 1.3, 1.9)	0.856	1.8 (0.9, 2.6)	0.231	0.2 (− 0.8, 1.2)	0.744		0.870
**Eotaxin**										
Baseline	0.8 (0.4, 1.3)				0.9 (0.6, 1.3)				0.694	
Wk 1	0.9 (0.2, 1.5)	0.958	− 0.1 (− 1.0, 0.9)	1.000	0.9 (0.5, 1.4)	0.990	0.0 (− 0.5, 0.5)	0.989		0.896
Wk 4	1.1 (0.8, 1.4)	0.464	0.2 (− 0.3, 0.8)	1.000	1.1 (0.7, 1.6)	0.421	0.3 (− 0.1, 0.7)	0.452		0.908
Wk 12	1.0 (0.1, 1.9)	0.829	0.3 (− 0.8, 1.3)	1.000	1.4 (0.9, 1.9)	< 0.0001****	0.6 (0.3, 0.9)	0.030*		0.401
**RANTES**										
Baseline	0.6 (0.3, 0.9)				0.7 (0.3, 1.1)				0.619	
Wk 1	0.7 (0.3, 1.0)	0.980	− 0.0 (− 0.4, 0.4)	0.999	0.5 (0.3, 0.7)	0.322	− 0.2 (− 0.6, 0.2)	0.335		0.493
Wk 4	5.4 (− 4.9, 15.7)	0.176	4.8 (− 5.5, 15.1)	0.176	0.8 (0.4, 1.2)	0.471	0.1 (− 0.3, 0.5)	0.650		0.285
Wk 12	0.7 (0.1, 1.4)	0.937	0.3 (− 0.4, 0.9)	0.880	0.9 (0.5, 1.2)	0.624	0.2 (− 0.2, 0.5)	0.722		0.738
**TNF-β**										
Baseline	0.2 (0.1, 0.3)				0.2 (0.1, 0.3)				0.921	
Wk 1	0.1 (0.1, 0.2)	0.532	− 0.0 (− 0.2, 0.1)	0.578	0.2 (0.1, 0.4)	0.529	0.1 (− 0.1, 0.2)	0.505		0.422
Wk 4	0.2 (0.1, 0.3)	0.504	0.0 (− 0.1, 0.1)	0.803	0.2 (0.1, 0.4)	0.544	0.1 (− 0.1, 0.2)	0.570		0.329
Wk 12	0.2 (0.0, 0.3)	0.543	− 0.0 (− 0.2, 0.2)	0.700	0.3 (0.2, 0.4)	0.105	0.2 (0.0, 0.3)	0.146		0.082

Values are presented as mean (95% CI).

*P < 0.05, **P < 0.01, ***P < 0.005, ****P < 0.0001.

^a^Generalized estimating equation compared with baseline.

^b^Cytokine change compared with baseline (generalized estimating equation).

^c^Comparison between baseline of ESWT and placebo (generalized estimating equation).

^d^Comparison between corresponding time point of cytokine change of ESWT and placebo (generalized estimating equation).

## Discussions

Similar to previous reports, ESWT was associated with a statistically significant decrease in OSS and VAS pain scale at 4 weeks post-treatment with lower intensity of placebo effect. In contrast to the previous report from our group^8^, placebo group has no statistically significant change in OSS and VAS pain scale at 4 weeks post-treatment, which is reflected in the detection of significant difference in symptomatic improvement between ESWT versus placebo that eluded us in previous study. The lower intensity of placebo effect in this study may be due to a more homogenous patient population in a single study center.

The main goal of the current study was to identify changes in urine biomarkers pre and post-treatment and investigation to the mechanistic understanding of ESWT efficacy on IC/BPS. It has been known that ischemia/hypoxia condition occurs in the bladder mucosa and contributes to IC/BPS symptoms^11^. VEGF is a signal protein that stimulates the formation of blood vessels to restore the oxygen supply to tissues when blood circulation is inadequate such as in hypoxic conditions. Bladder urothelium of IC/BPS patients has been shown to exhibit significantly higher expressions of VEGF, which then induces bladder fibrosis and reduces bladder capacity after chronic inflammation^11^. Furthermore, VEGF expression level was associated with the grade of bladder pain^12^. A previous study showed that intravesical botulinum toxin A injection reduced the expression of VEGF associated with a concomitant decrease in inflammatory marker levels in patients with IC/BPS^13^. Anti-vascular endothelial growth factor treatment has been demonstrated to decrease bladder pain in animal model of cyclophosphamide cystitis^14^. In the present study, urine VEGF level was significantly increased at 4 weeks follow-up in placebo group. However, the ESWT group showed a reduction of VEGF expressions at 4 weeks. Our study suggests that ESWT has the potential to decrease urinary VEGF expression and alleviate IC/BPS symptoms.

Sugaya et al. have reported that about 35% of the patients with interstitial cystitis had some type of allergic or autoimmune disease^15^, which is associated with overproduction of IL-4. IC/BPS is characterized by an increased number of mast cells in the detrusor and release of cytokines, including IL-4^16^. Our current results showed that IL-4 was significantly increased at 4 weeks in the placebo group, whose increase was suppressed by ESWT. We suggested that ESWT might have effects on immune modulation through mast cells IL4 reaction.

IL-9 is a cytokine secreted by *CD4* + *helper cells* that regulates a variety of *hematopoietic cells*, including stimulation of *cell proliferation* and prevention of *apoptosis*^17^. The current results showed that urinary IL-9 is increased in ESWT group, which was not observed in the placebo group at 4 weeks. This finding might indicate an immune modulation effect of ESWT on IC/BPS patients.

It is confusable to find that IL-1RA, IL-4, IFN**α2**, or VEGF elevated in the placebo group during the follow-up period. The dynamic character of disease activity or comorbidities might have influence on the urine biomarkers. It is possible that elevation of creatinine normalized levels of IL-1RA, IL-4, IFN**α2**, or VEGF in placebo group is not mechanistically linked to ESWT, but could be random error introduced by the normalization process. However, IL9 was significantly increased at week 1 and 4 post ESWT, and VEGF has a trend to decrease at week 1 and 4 post ESWT. Biomarker discovery in IC/BPS has been challenging, with considerable clinical effort and expense^18^. The current urine biomarkers data might generate a hypothesis to identify potential molecules linked to ESWT action for future study.

The rationale of this study is based on that (1) ESWT has anti-inflammatory, anti-apoptotic effects, (2) thus ESWT may be effective for IC/BPS that is known to be associated with an enhanced inflammatory responses, in conjunction with abnormal vascularity in the bladder tissue. The above suggested pathophysiologies such as enhanced immune responses, urothelial defect, abnormal vascularization, and dysregulated urothelial cell apoptosis are all for Hunner lesion IC (HIC), but not for IC/BPS without Hunner lesions (NHIC). The different forms of IC indeed represent completely different pathological entities, despite sharing similar symptomatology and the same chronic course. It has been reported that classic IC displayed a six to tenfold increase of mast cells while nonulcer IC revealed twice as many mast cells as controls^19^. Maeda et al. reported that substantial lymphoplasmacytic inflammation (≥ 200 cells/mm^2^) was observed in 93% of HIC specimens, whereas only 8% of NHIC specimens were inflamed^20^. A study of bladder mucosa specimens from 29 patients with IC/PBS (not limited to HIC) and 5 control patients showed the levels of pro-apoptotic proteins, including phospho-p53, Bad, Bax, and cleaved caspase-3 were significantly increased in the IC/PBS bladders^4^. Taken together, we suggested that NHIC might still have some level of inflammation, mast cells accumulation, and urothelium apoptosis, however, all of these pathophysiological findings were less severe compared to HIC. Therefore, we can observe some symptoms improved after ESWT in our patient population of NHIC.

The limitation of this study is the lack of a non-IC/BPS control arm and small sample size. The association of symptoms severity and variable urinary biomarkers in the IC/BPS patients are still undetermined and limited by large variability among subjects, impact of comorbidities, and lack of age-matched controls. Furthermore, the current study population has less comorbidies than the general IC/BPS patients, which might lead to selection bias from clinical study.

In conclusion, our clinical study demonstrated that compared to placebo, ESWT in IC/BPS patients improved OSS and pain scale in association with some urine cytokine and chemokine changes. Our study suggests that IC/BPS patients with elevated urine proinflammatory cytokines may be candidates for ESWT therapy. Further control study with larger sample size, and broader co-morbidities is necessary to elucidate the actual therapeutic efficacy and urine biomarker change of ESWT.

## Supplementary Information

Supplementary Information.
