# Supplementary material for: Improves symptoms and urinary biomarkers in refractory interstitial cystitis/bladder pain syndrome patients randomized to extracorporeal shock wave therapy versus placebo
Source: Sci Rep. 2021 Apr 6;11:7558. doi: 10.1038/s41598-021-87040-1 (PMC8024394; doi:10.1038/s41598-021-87040-1)
Supplement: Supplementary file 1 — Supplementary Information. [file 41598_2021_87040_MOESM1_ESM.pdf]

Improves Symptoms and **Urinary Biomarkers in** Refractory Interstitial Cystitis/Bladder Pain Syndrome **P**atients **Randomized to** Extracorporeal shock wave therapy **versus Placebo**

Yuan-Chi Shen[1, 2], Pradeep Tyagi[3], Wei-Chia Lee[1, 2], Michael Chancellor[4], Yao-Chi Chuang[1, 2]

Department of Urology [1], The Center of Excellence in Shockwave Medicine and Tissue Regeneration[2], Kaohsiung Chang Gung Memorial Hospital, Chang Gung University College of Medicine, Kaohsiung, Taiwan;
Department of Urology[3], University of Pittsburgh, School of Medicine, Pittsburgh, Pennsylvania
Department of Urology[4], Beaumont Health System, Oakland University William Beaumont School of Medicine, Royal Oak, MI

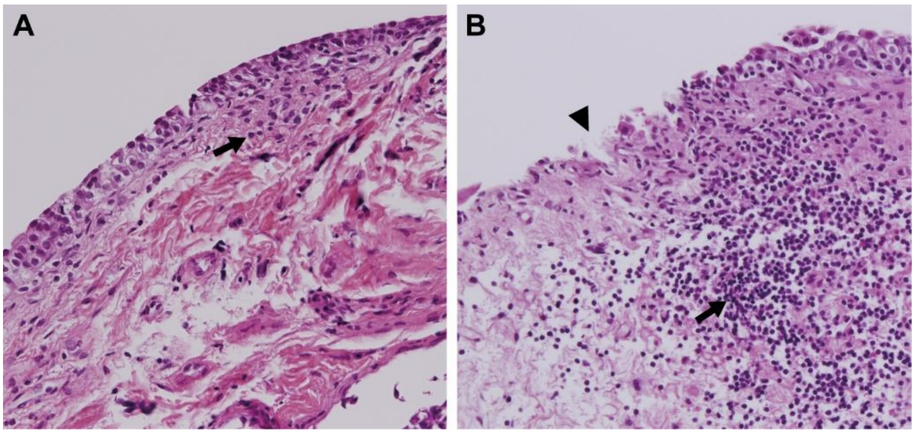

A. mild lymphocytic infiltration (arrow)
B. moderate lymphocytic infiltration (arrow) associated with mucosal denuding (arrowhead)
